# Scallop fishing activity characterization in Southern New England: Offshore wind demands and fisheries-dependent methods

**DOI:** 10.1371/journal.pone.0313197

**Published:** 2024-11-11

**Authors:** Julia Livermore, Todd Guilfoos

**Affiliations:** 1 Division of Marine Fisheries, Rhode Island Department of Environmental Management, Jamestown, RI, United States of America; 2 Department of Marine Affairs, College of the Environment and Life Sciences, University of Rhode Island, Kingston, RI, United States of America; 3 Department of Environmental and Natural Resource Economics, College of the Environment and Life Sciences, University of Rhode Island, Kingston, RI, United States of America; University of Messina, ITALY

## Abstract

Managers attempt to minimize spatial use conflicts in siting of offshore wind developments, but they must rely on available data to balance biological, commercial, and recreational needs. Marine spatial planning products are only as good as the data they are built upon and fishing data present major challenges due to their confidentiality and the difficulty in isolating true fishing activity. We present a methodology to increase the spatiotemporal resolution of fishing effort and exposure estimates for Southern New England scallop fishing activity using random decision forests to perform supervised classification on AIS data, with fallback to lower resolution datasets for vessels without AIS coverage. Final predictive accuracy of the tuned random forest AIS model was 97.9%, offering improvements of 24.7, 48.6, and 50% over VTR fishing footprints, and AIS and VMS speed cutoff methods, respectively, to predict whether vessel locations correspond to fishing activity. Comparison of the AIS model with VMS and VTR fallback to the VTR fishing footprints data product demonstrated that the increased precision of the AIS point data delineated as fishing dramatically changed how fishing effort, and therefore exposure in the form of fishery landings values, is distributed spatially in Southern New England wind energy areas. This is due to how the probability of fishing is distributed across location data points in the various products, which has implications for marine spatial planning and mitigation decision-making. Therefore, multiple data products should be considered when evaluating management options, as exposure estimates may differ depending on what inputs are used. The higher resolution AIS product may offer enhanced value in understanding exposure and impacts to individual vessels, especially once wind farms are under construction or operational.

## Introduction

Demand for ocean space in the United States exclusive economic zone has grown rapidly with the expansion of the offshore wind industry and increasing interest in federal waters for commercial aquaculture [1, 2]. Managers have attempted to minimize spatial use conflicts in siting of offshore wind developments, aquaculture farms, and other fixed spatial uses, but they must rely on available data to balance biological, commercial, and recreational needs [3–5]. Marine spatial planning products are only as good as the data they are built upon, and commercial fishing data present a major challenge to understanding how offshore areas are utilized due to their dynamic and confidential nature [6]. This is especially problematic because avoiding spatial use conflicts is difficult and so is determining the level of economic exposure and impact of changes to fishing activity in areas where conflicts do occur. Exposure is defined here as the fishing landings revenue that has the potential to be lost due to offshore wind development.

Very recently, the process of compensation for potential fishery losses in the form of lost revenue incurred due to fishing activity displacement has become a point of discussion in offshore wind development, as Vineyard Wind and South Fork Wind Farm’s fishery compensation programs opened in early 2024 [7–9]. While two programs are already actively processing claims of fishery losses, the allocations of funds were based on individual project and state-level negotiations with dramatically different approaches to determining fishery exposure and potential levels of development impact (e.g., [10–13]). For example, within the State of Rhode Island, fishery compensation negotiations between developers and the state (with fishing industry advisors) for Vineyard Wind and South Fork Wind focused on entirely different fishery-dependent datasets and used different methods to estimate possible levels of effect [10, 11]. Despite the inconsistencies across approaches, there remains a dearth of policy to guide (or require) and equitably distribute funds to affected fishers for a variety of reasons, including the lack of agreement on what commercial fishing data should be used and how it should be analyzed to determine fishery exposure.

Commercial fishing data for federally managed species in New England exist in a variety of forms, that include but is not limited to: dealer reports (also referred to as landings), vessel trip reports (VTRs), observer reports, vessel monitoring system (VMS) data, and the automatic information system (AIS). Each fishery-dependent data source was created with a specific purpose in mind (generally enforcement or management), but none were designed for the purpose of characterizing offshore commercial activity in terms of spatiotemporal distribution and economic value. Landings provide information on dollar value, amount, and grade of catch by vessel as it is sold to a dealer, but offers no information on the location of the corresponding fishing activity. For fishery management plans (FMP) that require them, VTRs do provide self-reported fishing locations, hail weights of catch, and effort information; however, each VTR provides a single point location of fishing activity (which may apply to an entire trip) [14]. VTRs may be used to attribute fishing effort on a more regional scale (e.g., Greater Atlantic Region Statistical Areas), but understanding fishing activity in potential development areas requires data at a significantly higher resolution (see [15] for a similar example). Observer reports can supplement VTRs by supplying verified locations and times of individual fishing tows or sets, but only cover a portion of fishing activity (e.g., [16]). VMS data offer higher resolution fishing location information (locations every 30 minutes or every hour depending on the FMP with additional caveats), but no information on catch [17]. Additionally, VMS resolution still falls short in many applications (e.g., [18–20]). The AIS presents even higher resolution location information (generally once per minute), but is only required on vessels greater than 65 feet in length [21] and location recording can be affected by satellite coverage or turned off by the vessel operator [22]. Further, all of the aforementioned data sources, except the AIS, are confidential data [23].

As a result, a variety of non-confidential data products have been developed by aggregating data into non-confidential formats or combining datasets in an attempt to better characterize fishing activity in federal waters. Considering most fishing datasets have limited spatial and temporal scope, combining data sources will be essential to develop a clearer understanding of fishing activity and exposure to developments [24]. However, all of these data products still have significant shortcomings due to the datasets that they were built upon. For example, DePiper et al. [25], Kirkpatrick et al. [26], and Benjamin et al. [27] use VTR data and ultimately must make assumptions about the location provided within the trip report. The National Oceanic and Atmospheric Administration’s (NOAA’s) Northeast Fisheries Science Center (NEFSC) and the Greater Atlantic Regional Fisheries Office (GARFO) estimate fishery exposure (defined here as the maximum value of lost ex-vessel value if no fishing occurs within a development area) using these DePiper et al. [25] and Benjamin et al. [27] methods to develop raster datasets of VTR fishing footprints [28]. These footprints ultimately portray the extent predicted from a single location provided in a trip report, rather than the true extent of the trip, which introduces bias into the exposure estimates; this bias depends on how much the fishing footprint location is restricted [29]. For example, for the squid fishery VTR-based fishing footprints may overestimate the number of vessels using a wind development area, but underestimate the impact to affected vessels [29]. Moreover, analysis and correspondence with commercial fishers conducted during mitigation discussions for the South Fork Wind Farm in Rhode Island through the Coastal Resources Management Council’s Fishermen’s Advisory Board suggested that fishermen tend to report on a VTR their first or last fishing location on a trip, or where they had the most catch. This means that the VTR data are likely to include reported locations that are biased towards the shoreline and away from wind development areas, leading to artificially low estimates of landings values within wind areas [30]. In contrast, Muench et al. [31] contend that fishing is most likely to be observed in the middle of a trip, since the beginning and end of a fishing trip are generally travel to and from port. This method is currently the primary tool used in fisheries mitigation discussions with wind developers (e.g., [11]) but it is not clear whether this approach has been adequately vetted by the scientific community [32].

Utilization of VMS or AIS data to describe offshore fishing activity presents a related, but distinct issue. Palmer and Wigley [33] suggest that VMS polls with an imputed speed between 3.7 and 7.4 km/h (2–4 knots) for otter trawls, 4.6 and 11.1 km/h (2.5–6 knots) for scallop dredge and 0.2 and 2.4 km/h (0.1–1.3 knots) for sink gillnet are usually fishing. Lee et al. [34] contend that a speed range of 1–8 knots, independent of gear type, identifies fishing pings in VMS data. Based on commercial fishing industry input, public VMS data products on the Northeast Regional Ocean Data and Mid-Atlantic Ocean Data portals utilize a 4-knot speed cutoff for most fisheries, and a 5-knot cutoff for scallop dredge, to generally delineate fishing activity from transiting [35]. However, Muench et al. [31] demonstrate that representation of fishing activity that has been derived using speed rules leads to severe misrepresentation of fishing for most gears, with the exception of bottom otter trawling. Ultimately, a basic speed filter is more biased to false positives than false negatives, leading to misallocation of fishing activity to non-fishing locations. As such, data products based on VMS or AIS where fishing is delineated based on speed may not accurately be attributing fishing to vessel activity; this includes the data products on the regional ocean data portals [35], as well as more advanced methods linking VMS to landings data (e.g., [36]) and AIS products (e.g., [37]). Despite these challenges in delineating fishing versus non-fishing activity using a speed filter, the higher resolution of the VMS data inputs still generates more spatially explicit data products than relying on VTR alone.

O’Farrell et al. [38] developed a machine learning approach to address this specific issue using feature engineering by changing the way location pings are labeled when training the model. Instead of labeling fishing points individually, they developed a method using window labeling to engineer model features, where VMS records were labeled as fishing if gears were deployed within the hourly ping window surrounding that ping, rather than recording whether fishing was occurring only at the timestamp the ping was logged. The window labeling approach dramatically improved model true-positive/balanced accuracy in prediction of fishing activity in VMS data. Other methods developed to separate fishing from non-fishing activity in AIS and VMS datasets include, but are not limited to, recursive Bayesian filtering procedures [39], boosted regression trees [40], layered filtering approaches [20], hidden Markov models [41], and convolutional neural networks [42].

These papers all advance the methods in delineating fishing and non-fishing activity in vessel location datasets, which may enable usage of higher resolution datasets in understanding the spatial distribution of fishing effort. Allen-Jacobson et al. [29] highlight the value of fine-scale fishing data for understanding fisheries exposure to offshore wind development since higher resolution data are available for many fisheries in Southern New England in the form of AIS, similar methods could be utilized to improve fishing prediction accuracy.

Here we present a comprehensive approach to characterizing offshore fishing activity incorporating machine-learning on fine-scale AIS data to delineate fishing versus non-fishing activities, with fallback to existing VMS and VTR approaches for vessels without AIS.

## Materials and methods

### Data

**Fig 1:**
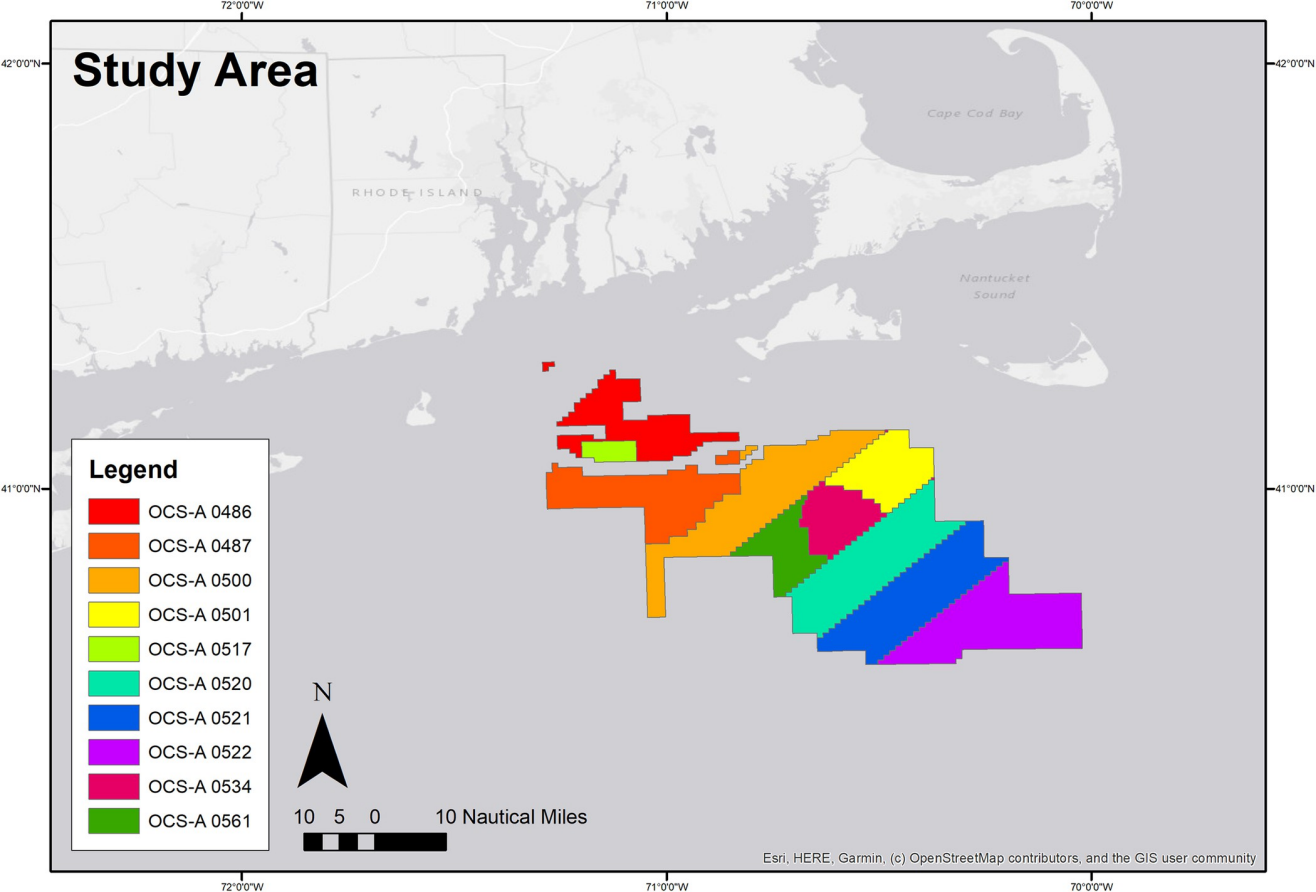
Study area. Southern New England (SNE) offshore wind lease areas (as of July 3,2024) are also shown.

All data sources were acquired for the years of 2015–2018 based on the years that all datasets were available at the time of request or download for the study area (Fig 1). To establish an appropriate methodology, analysis was focused on a single fishery management plan: the Atlantic scallop fishery. Publicly available AIS data were downloaded from Marine Cadastre (https://marinecadastre.gov/ais/). Confidential VMS data were requested through NOAA’s Office of Law Enforcement and received after execution of a non-disclosure agreement. Confidential trip-level VTR data and dealer reports were obtained through the Atlantic Coastal Cooperative Statistics Program’s (ACCSP) Data Warehouse. Public GARFO permit files were downloaded from https://www.greateratlantic.fisheries.noaa.gov/public/public/web/NEROINET/aps/permits/data/index.html. Confidential Northeast Fisheries Observer Program (NEFOP) data were requested and received through NOAA’s NEFOP program. The observer dataset includes start and end locations and timestamps of all tows, as recorded by an independent on-board fisheries observer. Bathymetry data were downloaded from the Northeast Ocean Data Portal: https://www.northeastoceandata.org/files/metadata/Themes/Bathymetry/Bathymetry.htm. Moon phase data were compiled from monthly calendars available at https://www.almanac.com/astronomy/moon/calendar. Modeled VTR location data (an intermediary step of Benjamin et al. [27] were obtained from the Northeast Fisheries Science Center (NEFSC). An ESRI feature class of offshore wind lease areas was downloaded from the Bureau of Ocean Energy Management (BOEM) at https://www.boem.gov/renewable-energy/mapping-and-data/renewable-energy-gis-data.

### Methodology

#### Data processing

**Fig 2:**
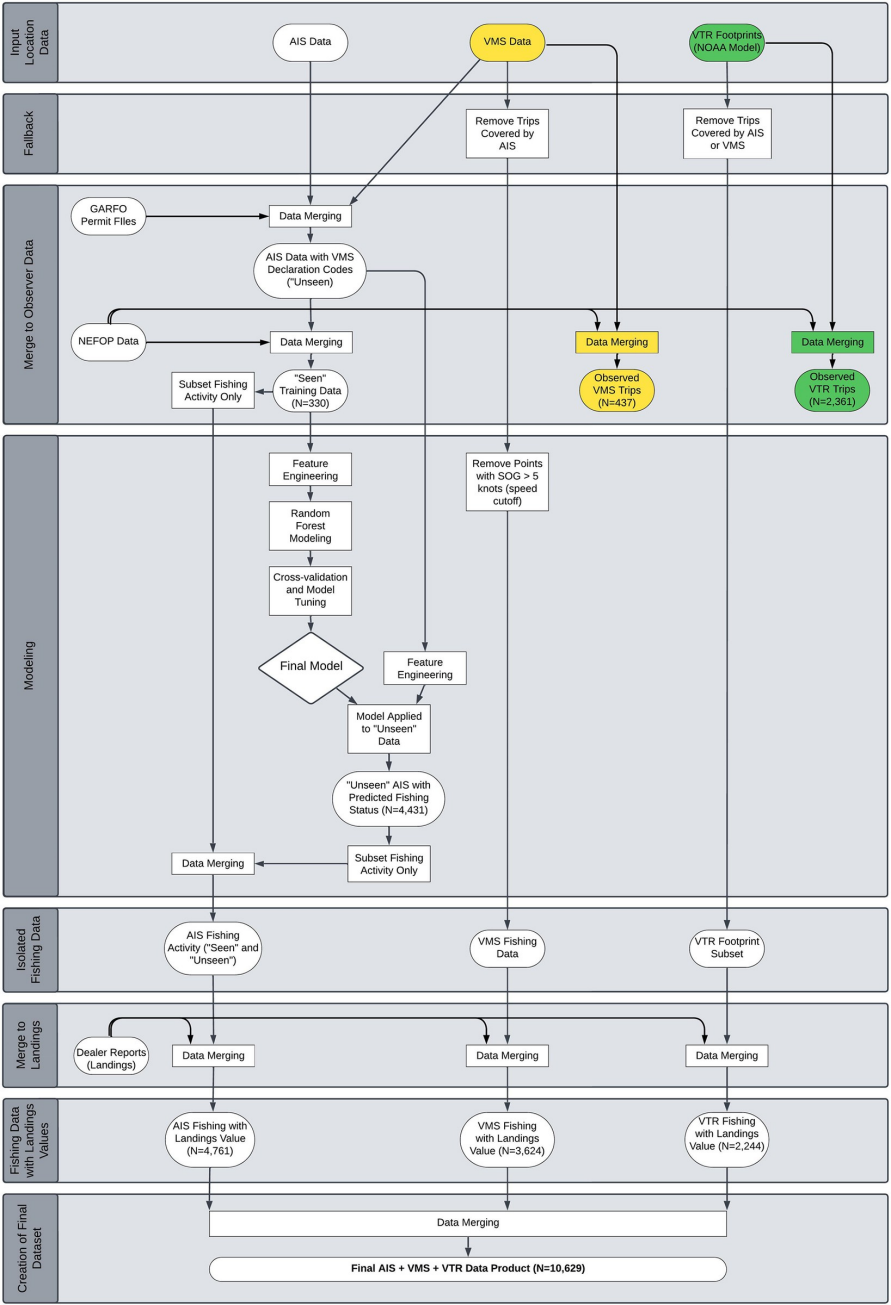
Methodology flowchart. Ovals represent datasets including inputs, intermediary steps, and outputs. Rectangles are processes (e.g., data merging, modeling steps). White shapes depict the process of building the final AIS data product. Shapes shown in yellow or green are also included in creating data products used for comparison to the AIS model’s predictive accuracy, where yellow corresponds to the VMS comparison product and green corresponds to the VTR comparison product. Sample sizes shown for datasets are the number of fishing trips included within that dataset.

AIS data were first linked to GARFO permit files to determine vessel fishing permits (Fig 2). The merged AIS data were also linked to VMS data to identify the fishery management plan the trip was operating under through the VMS declaration code. All AIS locations within state waters were omitted from further analysis. To generate training data, AIS data were then merged to NEFOP data to mark all AIS pings that corresponded with fishing (i.e., timestamp fell between start and end of an observed haul). To correct variances in time before engineering features, all data were resampled on 1-minute intervals using linear interpolation between points for relevant fields. Finally, features were engineered on a 15-minute rolling window for the following features: average speed over ground, standard deviation of speed over ground, straight line distance (geodesic) from start to end locations, total distance (geodesic) traveled, average depth, standard deviation of depth, average of change in course over ground between consecutive points, and change in course over ground from start to end locations. Additional features included are moon light (percentage), month, and day of the week. The final training dataset after merging included 143 vessels on 330 separate trips, resulting in 8,487 individual observed hauls and 2,770,714 location pings, where a trip is all activity the vessel engaged in between leaving and returning to port and a haul is the activity while fishing gear is in the water and corresponding catch being hauled and sorted on deck.

#### Modeling

Supervised classification was conducted in Python (Python Software Foundation. Python Language Reference, version 3.11. Available at http://www.python.org) using the Skicit-learn library [43]. Random decision forests were selected because they present an option to overcome the limitations of modeling non-linear relationships, since decision trees model data hierarchically and branch data into leaves that represent predictions. Unconstrained decision trees can also easily be overfit but methods like random forests ensemble trees to limit overfitting [44]. Further, Behivoke et al. [45] found that random forest modeling was the most reliable method for processing global positioning system (GPS) tracks and identifying spatially-explicit fishing activity, independent of gear type.

Random forests ensemble a large number of relatively unconstrained decision trees to smooth out the predictions. They work by creating a bootstrapped sample set from the training dataset, growing a random forest decision tree for the bootstrapped data, and averaging the outputs of all the individual trees. Hastie et al. [44] and Yoon [46] offer detailed descriptions of the random forest algorithm, summarized as follows:

1. For *k* = 1 to K:
  - Pull a bootstrap sample Z* of size *N* from the training dataset.
  - Grow a random forest decision tree *T*_*k*_ to the bootstrapped data by recursively repeating the steps below for each terminal node on the tree, until the minimum node size *n*_*min*_ is reached:
    - Select *m* variables at random from the *p* variables.
    - Pick the optimal variable/split-point among the *m*.
    - Split the node into two daughter nodes.
2. Output the ensemble of random forest trees ${\left\{T_{k}\right\}}_{1}^{K}$.

To make a prediction at a new point *x*:

*Regression*: ${\hat{f}}_{rf}^{K}(x)=\frac{1}{K}\sum_{k=1}^{K}T_{k}(x)$

*Classification*: Let ${\hat{C}}_{k}(x)$ be the class prediction of the *k*th random forest tree. Then ${\hat{C}}_{rf}^{K}(x)$ = *majority vote* ${\left\{{\hat{C}}_{k}(x)\right\}}_{1}^{K}$.

Cross-validation was used to tune model hyperparameters including number of trees and tree depth as well as to select the number of model features to include. Feature selection was done based on recursive feature elimination during cross-validation and feature importance. O’Farrell et al. [38] note that out-of-bag (OOB) error rate often replaces cross-validation in random forest applications to classification. OOB error stabilization was used to evaluate overall model performance because it provides an unbiased estimate of model performance since it is calculated on out-of-bag samples unseen by the model. It can also happen simultaneously with model fitting making it computationally efficient [44].

The trained model was applied to the unseen AIS dataset and then merged to landings data to allow for ex-vessel value to be distributed across fishing locations. Within each trip, values of sold catch were evenly distributed among all fishing locations in the AIS dataset. Other methods of distribution were considered, including using annual vessel density by location or using interpolated fishery-independent scallop abundance data to weight values within a trip, but both approaches introduced added uncertainties. For this reason, an objective approach of distributing value across points equally was taken.

#### Fallback to VMS and VTR

For any trips in the VTR dataset without corresponding AIS data, VMS data were used instead, if available. VMS data were merged with VTRs and landings to isolate relevant activity, and cross checked with AIS to avoid double counting. Locations within the VMS dataset were parsed into fishing and non-fishing, based on a speed cutoff of five knots, per Fontenault [35]. Landings values were then distributed evenly across fishing locations, as was done with the AIS dataset. Trips with no corresponding AIS or VMS relied on modeled VTR locations. For each individual reported VTR location, a raster of modeled distribution of fishing probability (totaling to 1.0) was provided by the NEFSC; these rasters are used to create NOAA’s fishing footprints data projects. These rasters were selected by trip number to avoid duplication with the AIS or VMS datasets, multiplied by ex-vessel value of landings of the trip, and were then summed over individual years using basic raster math.

#### Combining datasets

AIS and VMS point datasets were combined into a single dataset and then rasterized into a 500 m x 500 m raster grid (matching the resolution of Benjamin et al. [29]), where raster values correspond to the sum of point values (ex-vessel dollars) within each grid cell. This grid was then added to the VTR grid using raster math to create the final raster of landings value by location.

#### Comparison to other data products

To assess how well the AIS model performed against existing data products, a variety of methods were employed. First, a 5-knot speed cutoff was applied to the AIS data for trips with NEFOP coverage and then the proportion of locations where the prediction was correct was calculated. Second, for VMS data with corresponding NEFOP coverage, tow start and end times were used to parse whether the vessel was fishing or not fishing at each location. Then the speed cutoff approach was applied and predictions were compared to the NEFOP verified fishing status. Finally, VTR fishing footprint data (the modeled distribution of fishing probability totaling to 1.0) for each individual trip with observer coverage was compared against a raster of matching size and resolution containing NEFOP verified haul location information for that trip. The haul location raster was created by plotting each haul as a line from start to end and converting the vector data to a raster where cells intersected with a haul receive a value of 1 and cells with no fishing activity were assigned a value of 0. This raster was then compared to the trip’s VTR fishing footprint using ordinary least squares regression. This was all VTR trips with observers onboard, generating 2,361 models. In order to compare the final aggregated AIS, VMS, VTR product against the VTR fishing footprint data, the difference between the two output rasters for the full time period was calculated.

The full combined AIS model with VMS and VTR fallback raster dataset was also compared against the VTR raster layer for all trips covered in both datasets. Raster values represent spatially aggregated scallop fishery exposure estimates. Raster values were summed within wind lease areas to compare exposure estimates. Kernel density estimates of raster values were also created for individual lease areas for both data products.

**Fig 3:**
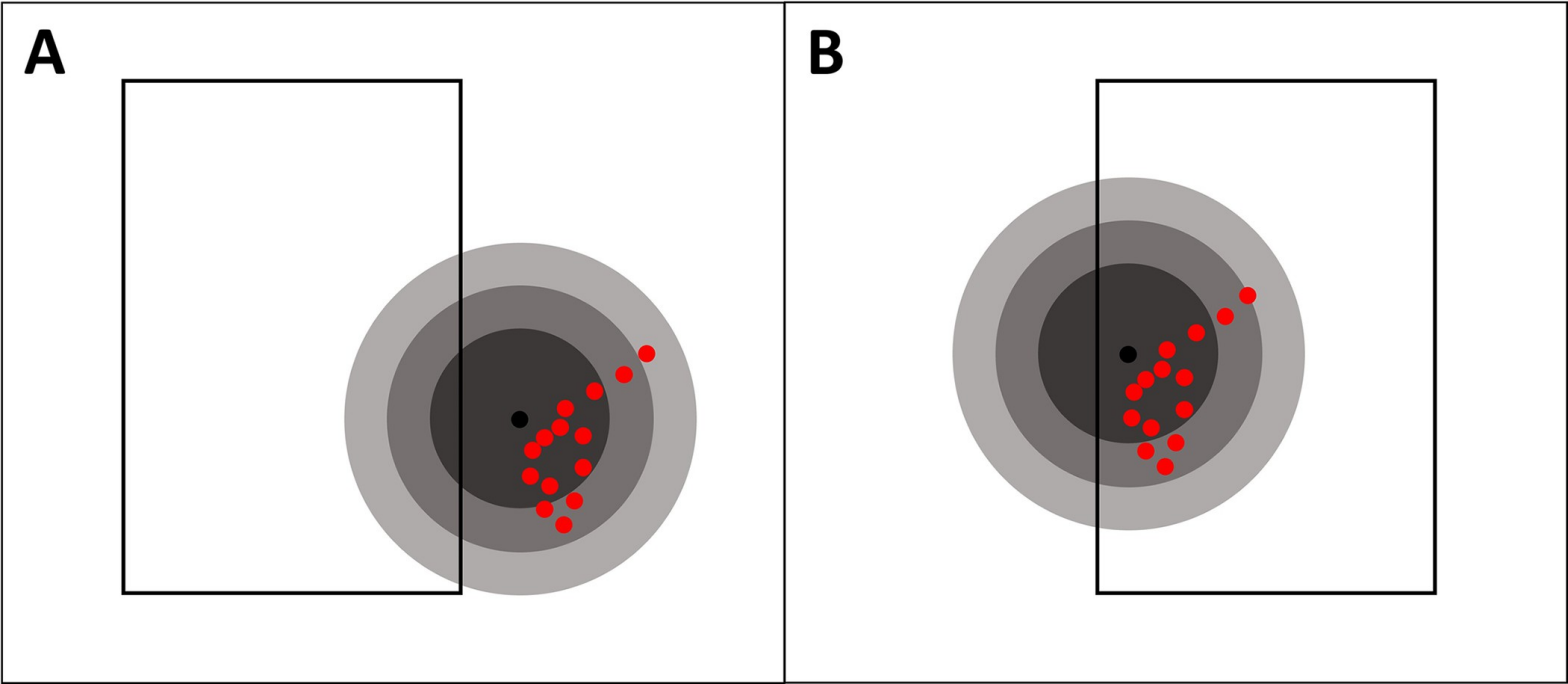
Assignment of trips to lease areas. Black outlines represent lease areas. Black dots are self-reported VTR locations, with concentric circles distributing landings values based on modeled probabilities of fishing location (per Benjamin et al. [27]). Red dots are AIS pings delineated as fishing by the AIS model.

Since the VTR model distributes landing value spatially around a single reported location, and the AIS model distributes value only to actual vessel locations, the AIS approach is likely to distribute value more tightly. This could potentially generate mismatches where the VTR approach is either misattributing value to a lease area or outside of a lease area (Fig 3). To address this analytically, an intrusion analysis was conducted for all trips with AIS coverage and corresponding VTR fishing footprints. For each trip, the AIS fishing values were overlaid on lease areas to determine what value corresponded to each lease area, or outside of the leases. The same was done for the VTR model for that same trip. The estimates between the two were compared by calculating the difference between the two (subtracting the AIS estimate from VTR estimate) for each individual lease. Trips where estimates within lease areas differed between the two models were isolated and differences were analyzed further with summary statistics and a kernel density plot.

#### Data coverage

After compiling all the primary datasets, coverage by AIS, VMS, and VTR was calculated for generating the comprehensive data product (Table 1). Overall VMS and VTR datasets covered more fishing trips than what is shown here, as AIS was used as the target dataset. A stepwise triage was conducted by dataset where AIS was the primary dataset with “fall back” to VMS, and then to VTR only when necessary. Within the AIS dataset, observers were present for 330 of the trips, equating to a NEFOP coverage rate of 6.93%. This data was used as the model training dataset, the “seen” dataset, and included 2,770,714 recorded locations for 143 fishing permits.

**Table 1:** Coverage of scallop fishing activity within the study area by data source.

Source	# Trips	% Coverage	# Seen Trips	% Seen
AIS	4,761	44.79%	330	6.93%
VMS	3,624	34.10%	NA	NA
VTR	2,244	21.11%	NA	NA

Total trips = 10,629. Seen trips correspond with trips with AIS coverage for which a fisheries observer was present. Observers were also present on some trips with VMS or VTR coverage, but these coverage rates were not assessed for this study.

## Results

### AIS model performance

After random forest model tuning (see S3 and S4 Figs), 10 features were ultimately selected to be included:

- Crow_flies_km (kilometers traveled in a straight line)
- Depth_Std (standard deviation in depth)
- Depth_Avg (mean of depth)
- SOG_Avg (mean of speed over ground)
- SOG_Std (standard deviation of speed over ground)
- COG_Avg_Abs_d (mean of change in course over ground, also called heading)
- d_COG_StartEnd (change in course over ground from the start point to the end point)
- Moon (moon phase as a percentage for that date)
- Day of the week
- Month

Consider that most features were engineered over 15-minute windows, where the calculated feature was a measure within that 15-minute time period. For example, the standard deviation feature was the standard deviation within the 15 minutes around the target point using a moving window approach; this particular feature was intended to identify whether a vessel was changing speed or moving at the same speed during the temporal window. The standard deviation in speed emerged as the most important variable, followed by distance traveled in a straight line, standard deviation of depth, mean of depth, and average of speed. The final model produced predictions with 97.9% accuracy and an out-of-bag error of 0.021.

### Comparison to other data products

As stated previously, the prediction accuracy of the AIS random forest model was ~98%. For comparison to accuracy of VMS speed-cutoff methods, the 5-knot cutoff (>5 knots indicates non-fishing activity) applied to the training AIS dataset correctly predicted fishing status for 49.3% of recorded locations. Results were similar when compared to the VMS. For trips with VMS and NEFOP observers on board, the speed cutoff approach was correct for 47.9% of recorded locations. VTR fishing footprint fishing prediction accuracy based on OLS regression, calculated as the mean of the coefficient values for the sole model coefficient in all trip-level models, was 73.2%.

**Fig 4:**
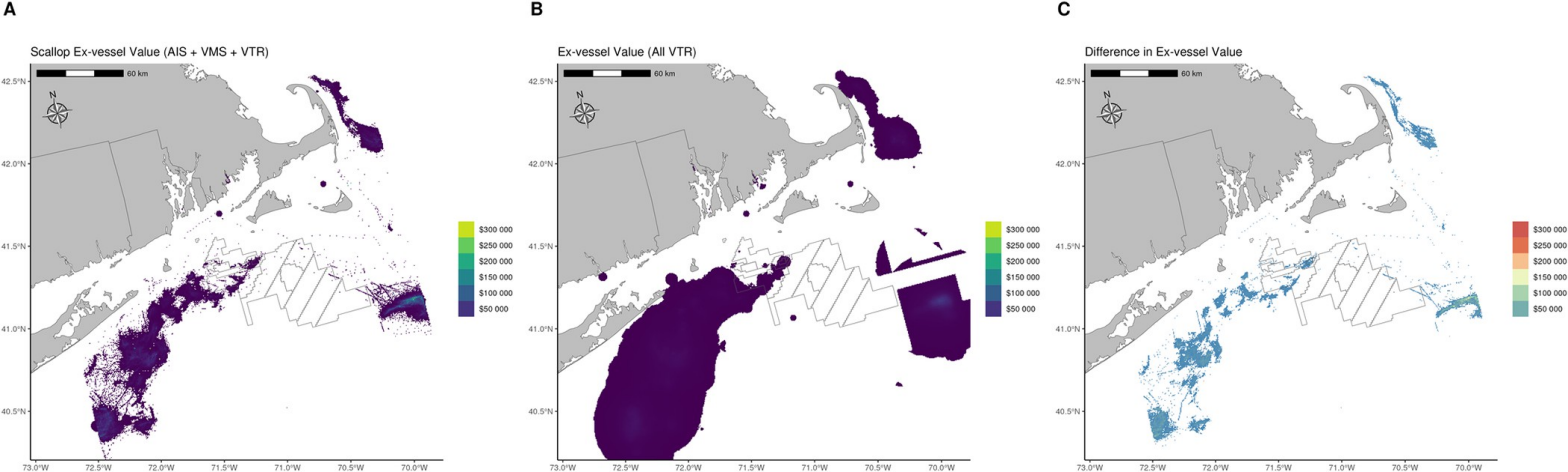
Final scallop exposure data products for 2015–2018. (A) AIS model with VMS and VTR fallback. (B) VTR fishing footprints provided by NEFSC for comparison. (C) The difference calculated between the two rasters. All rasters show only values greater than $500 to aid visual interpretation.

Exposure estimates within SNE lease areas differed between the AIS model with VMS and VTR fallback and the VTR fishing footprints data products (Fig 4). For most years in most lease areas, the VTR fishing footprint exposure estimate was larger than the AIS with fallback estimate; this was not always the case (Table 2).

**Table 2:** Aggregated annual and total dollars of scallop landings from SNE wind lease areas based on AIS model with VMS and VTR fallback versus VTR fishing footprints.

Lease Number	Year	$ derived from AIS model with VMS and VTR fallback	$ derived from VTR Fishing footprints	Difference in Estimates
OCS-A 0486	2015	$47,909	$118,330	-$70,422
	2016	$91,457	$122,536	-$31,079
	2017	$11,157	$39,910	-$28,753
	2018	$1,312	$13,071	-$11,759
	Total	$164,497	$293,848	-$129,351
OCS-A 0487	2015	$388,494	$355,295	$33,199
	2016	$422,497	$503,061	-$80,563
	2017	$201,628	$296,523	-$94,895
	2018	$13,965	$36,835	-$22,870
	Total	$1,047,252	$1,191,713	-$144,462
OCS-A 0500	2015	$29,933	$139,211	-$109,278
	2016	$34,898	$111,341	-$76,444
	2017	$4,629	$54,397	-$49,768
	2018	$5,509	$21,431	-$15,923
	Total	$79,253	$326,380	-$247,127
OCS-A 0501	2015	$49	$8,471	-$8,422
	2016	$3,543	$13,888	-$10,345
	2017	$780	$2,504	-$1,724
	2018	$27,287	$2,067	$25,219
	Total	$40,679	$26,931	$13,749
OCS-A 0517	2015	$5,173	$19,984	-$14,811
	2016	$649	$19,089	-$18,440
	2017	$3,455	$13,787	-$10,332
	2018	$182	$3,243	-$3,061
	Total	$10,324	$56,103	-$45,780
OCS-A 0520	2015	$46	$20,915	-$20,868
	2016	$1,725	$18,772	-$17,048
	2017	$42	$3,846	-$3,803
	2018	$8,087	$8,961	-$874
	Total	$25,940	$52,493	-$26,554
OCS-A 0521	2015	$0	$23,490	-$23,490
	2016	$0	$19,261	-$19,261
	2017	$107	$4,332	-$4,225
	2018	$68,747	$36,867	$31,880
	Total	$90,215	$83,950	$6,265
OCS-A 0522	2015	$47,909	$118,330	-$70,422
	2016	$91,457	$122,536	-$31,079
	2017	$11,157	$39,910	-$28,753
	2018	$1,312	$13,071	-$11,759
	Total	$164,497	$293,848	-$129,351
OCS-A 0534	2015	$388,494	$355,295	$33,199
	2016	$422,497	$503,061	-$80,563
	2017	$201,628	$296,523	-$94,895
	2018	$13,965	$36,835	-$22,870
	Total	$1,047,252	$1,191,713	-$144,462
OCS-A 0561	2015	$29,933	$139,211	-$109,278
	2016	$34,898	$111,341	-$76,444
	2017	$4,629	$54,397	-$49,768
	2018	$5,509	$21,431	-$15,923
	Total	$79,253	$326,380	-$247,127

Lease numbers correspond to the offshore wind leases shown in Fig 1. These numbers should not be used directly in offshore wind planning or mitigation; this exercise was meant to compare methods and not all applicable trips may be included in the final outputs.

**Fig 5:**
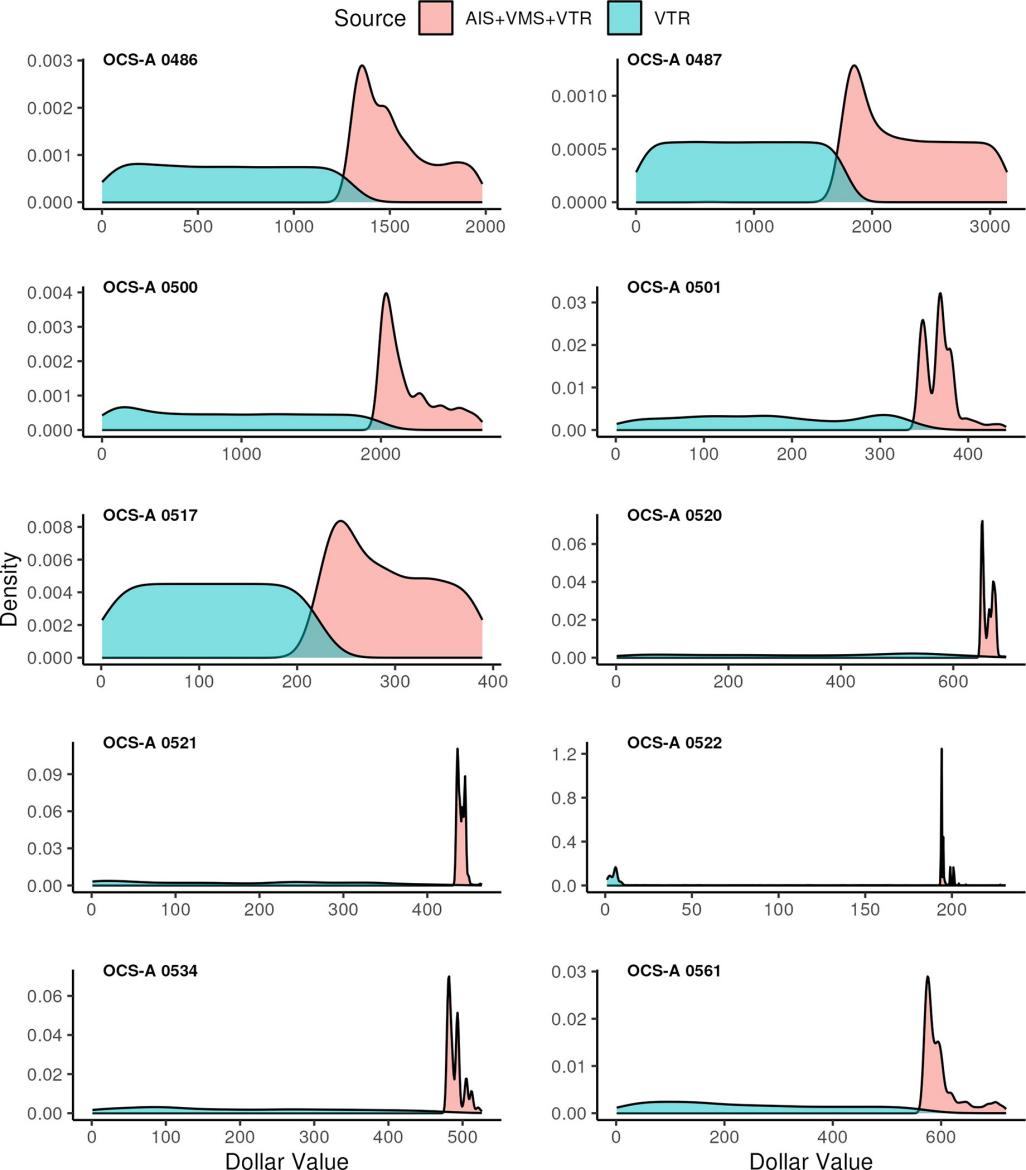
Kernel density estimates of raster cell values from AIS model with VMS and VTR fallback versus VTR fishing footprints for each of the Southern New England lease areas. Raster exposure values were aggregated over the 2015–2018 study period.

Kernel density estimates by lease area for the two data products demonstrate that scallop landings are distributed differently by the two methodologies (Fig 5). Raster values in the aggregated AIS model with VMS and VTR fallback were generally higher than values from the VTR fishing footprints; this was seen in all ten wind lease areas assessed.

**Fig 6:**
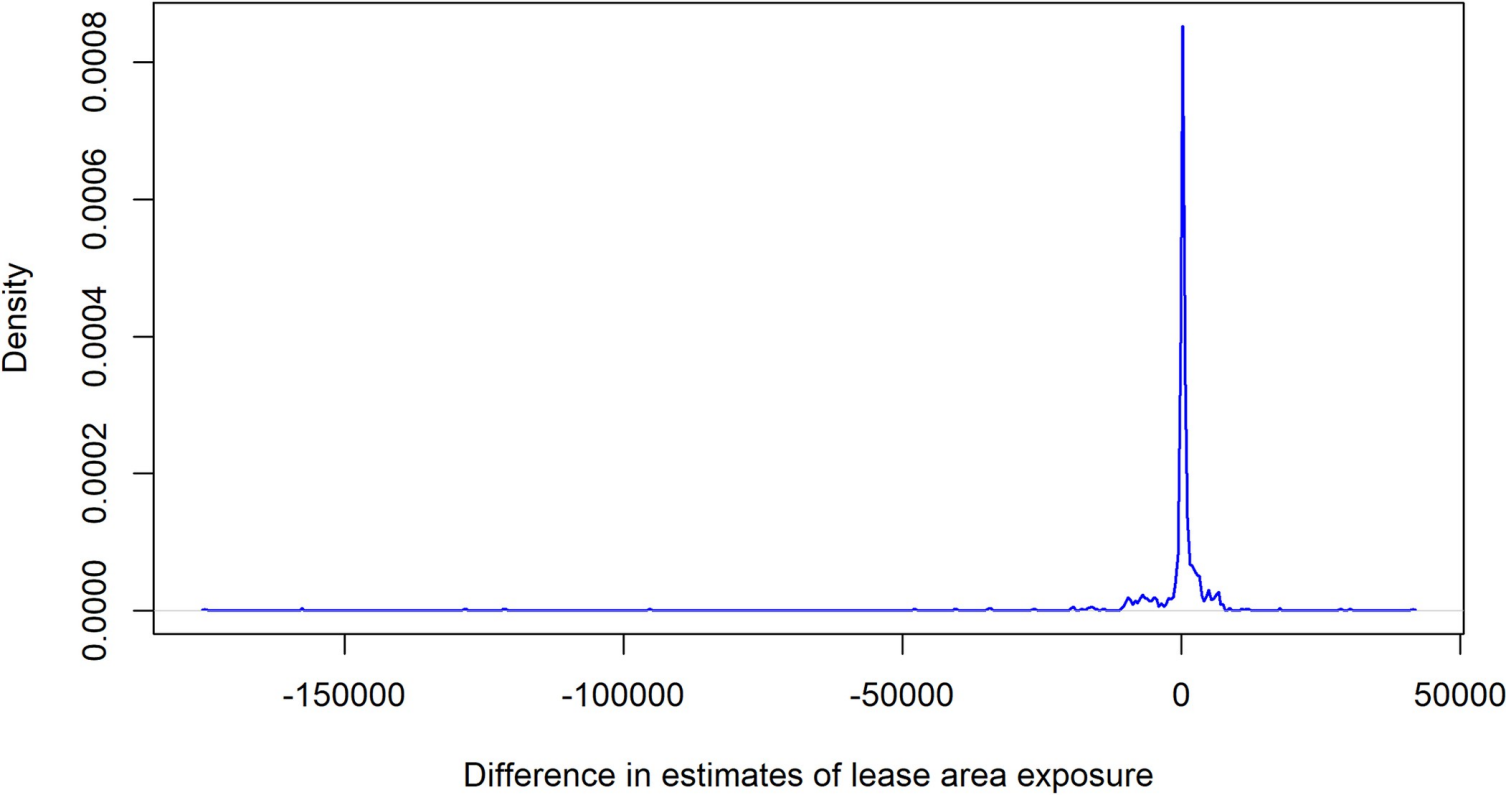
Kernel density plot of differences in VTR and AIS estimates. Differences calculated between the VTR exposure estimate and AIS model exposure estimate in lease areas for all individual trips with AIS coverage from 2015–2018; trips with matching estimates were omitted. Differences calculated as VTR estimate minus AIS estimate; positive values are indicative of the VTR estimate being larger.

Based on intrusion analysis, 85.7% of trips had the same estimate between the two approaches, while 14.3% of trips had disparities between the two for at least one of the 10 wind lease areas. For the trips with differences in exposure estimates, the mean of the differences was -$1,605.50, while the median was $154.72 per trip. The standard deviation was ± $14,886.32. Considering that differences were calculated as VTR estimate minus AIS estimate, the mean indicates that AIS estimates were larger, while the median suggests that the VTR estimates were larger. While trip-level VTR estimates were generally slightly larger than AIS estimates, the distribution of estimates is heavily negatively skewed due to a number of trips where AIS estimates were dramatically larger than VTR estimates (Fig 6).

## Discussion

As stated earlier, most fishery-dependent data are collected for the purpose of fishery management or enforcement, and may not be sufficient for offshore wind planning or understanding potential impacts [29, 36]. As such, a variety of fishery-dependent data products have been developed to increase suitability for understanding offshore development and fishing activity overlap (e.g., [27, 28, 36]). Nevertheless, each data product still has shortcomings due to the input data sources (e.g., spatiotemporal resolution) and necessary assumptions made during analysis (S1 Table). Using a random forest machine learning approach, this effort aimed to use fine-scale AIS data to expand upon existing fishery-dependent data products based upon coarser-scale inputs, such as VTR and VMS.

### Differences in data products

The AIS model demonstrated dramatic improvements in predicting fishing activity accurately as compared to both VMS and VTR data products. AIS model predictions exceeded 97% accuracy, while speed cutoffs applied to AIS and VMS predicted fishing accurately only 49.3% and 47.9%, respectively, and VTR fishing footprints predicted fishing activity 73.2% of the time. The AIS model approach offered improvements ranging from 24.7% and 50% improvement in model prediction accuracy over existing approaches. However, the limited vessel coverage rate makes the AIS data alone less useful. This work demonstrates the improved accuracy in using AIS and machine learning and offers a method to couple the AIS model with the next best available data for each individual trip, resulting in the most accurate, comprehensive fishing exposure estimates.

This effort has highlighted the shortcomings of using speed alone to delineate fishing versus non-fishing activity in the VMS, as discussed by Muench et al. [31]. Further, the outputs of the AIS with fallback approach resulted in a more precise distribution of fishing landings values offshore as compared to the VTR fishing footprints model due to the higher resolution of the primary dataset, and the reduced need to estimate vessel location at the time of fishing.

The VTR fishing footprint data product has been used as the primary tool used by offshore wind developers in fishery exposure estimates for project Construction and Operations Plans (e.g., [47]). Allen-Jacobson et al. [29] discuss the value of the VTR fishing footprints at the 90th percentile for capturing all trips with exposure to development of an area. This effort’s comparison to the AIS model with VMS and VTR fallback demonstrated that the increased precision of the AIS point data delineated as fishing dramatically changed how fishing effort, and therefore exposure in the form of fishery landings values, is distributed spatially. The VTR fishing footprint product distributes landings around a reported fishing location based on modeled probability of fishing [27], which results in a smoothed distribution of fishing effort as seen in Fig 5. The AIS model approach does not distribute fishing effort, but rather parses fishing from non-fishing ping locations, and then falls back to VMS and VTR, resulting in more tightly distributed estimates of fishing effort (Fig 5).

Interestingly, the differences in landings distribution between the two data products is not consistent across years or lease areas (Table 2). Estimates in the VTR fishing footprints product were generally higher, but not in all years or wind lease areas. The higher VTR estimates make sense, as the fishing footprint approach will create overlap with wind lease areas when fishing activity may have occurred just outside a lease area, which the AIS-based product may classify this fishing as outside the lease area (Fig 3). In contrast, there are other instances where fishing may have occurred within a lease area that the AIS-product correctly quantifies the landings value for, while the VTR fishing footprints may distribute some of the landings to outside the lease and the VTR point itself may be outside the lease.

Intrusion analysis confirmed this to be the case, where some trips had larger VTR exposure estimates and others had larger AIS exposure estimates within lease areas (Fig 6). For trips where the models distributed fishing values differently in lease areas, VTR estimates were generally larger than AIS estimates, but only slightly larger (e.g., the median was a $154.72 difference). For those trips where the AIS estimate was larger, it was substantially so (in some cases exceeding $150,000). Therefore, in aggregate, the VTR estimates were more conservative, while AIS estimates highlighted some major discrepancies between approaches at the individual trip level. Our findings align with those of Allen-Jacobson et al. [29], where lease level estimates using the VTR footprints may overestimate total exposure, but underestimate trip-level exposure based on comparisons to higher resolution study fleet data for longfin inshore squid (*Doryteuthis pealeii*) fishery.

In short, the AIS model substantially improves upon the accuracy of predicting fishing activity in vessel location datasets, but provides lower coverage than the VTR dataset. Therefore, the AIS model needs to be combined with other datasets to ensure full coverage of the fishery. The comprehensive approach here uses the best-available data for every individual trip, improving overall accuracy. However, building this product is complex (i.e., Fig 2) and computationally demanding. In contrast the VTR fishing footprints product offers high coverage of trips and is a readily available tool through NOAA [28].

### Recommendations

The increased precision of the final combined AIS data product enhances the usefulness of this product over VTR- and VMS-derived products for project micrositing, and may offer a more detailed understanding of fishing effort during fishery mitigation and compensation discussions, especially at the vessel- or trip-level. However, it offers limited added value for general project siting and overall project exposure estimation because the same general areas of fishing are identified by both products. Using VTR fishing footprints at the 90th percentile may therefore offer the most conservative approach to initial spatial planning efforts because it distributes fishing effort beyond the reported locations. Moreover, the differences in estimates across fishery-dependent data products demonstrate the value of assessing multiple sources of fishing effort data in offshore wind development and more general marine spatial planning decision making.

This study focused solely on distributing fishing effort and ex-vessel values (the value of catch sold to the dealer when a vessel lands). No considerations were made for possible shore-side economic impacts to the scallop fleet. Further, fishing grounds may shift in the future in response to changing ocean conditions, independent of offshore wind development [48]. Managers should consider multiple data streams, with an understanding of their various caveats, in addition to these additional factors to arrive at informed decisions.

It is not well understood how harvesters will respond to offshore wind farms during construction or once operational [49]. Studies that address this topic exist in other regions (e.g., [50, 51]), but the fisheries and their specific vessel and gear configurations in the Northwest Atlantic present new variables. However, the AIS with VMS and VTR fallback data product offers substantial advantages in assessing the response of harvesters to wind development. The combined AIS data product will allow for detailed assessment of if and how vessel activity changes in response to offshore wind development, and will offer the ability to measure vessels’ proximity to wind infrastructure installed offshore (e.g., turbine foundations, cables, or substations). Such data will enable an enhanced understanding of the impacts of wind development on fisheries in Southern New England and create new insight to improve future wind planning and fisheries management efforts.

Given the various differences between the combined AIS data product and the VTR fishing footprints, this work stresses that multiple data streams should be considered in all aspects of offshore wind planning and mitigation. VTR footprints at the 90th percentile are accessible for multiple years and fisheries and offer a conservative approach to identifying areas for proposed development, while the combined AIS data product may be more accurate for use in assessing individual vessel exposure and response to offshore wind development.

## Supporting information

S1 Table Data acquisition. (PDF)

S1 Fig Model AUC score as a function of the number of estimators (RF trees). This step was used during model hyperparameter tuning to select the number of estimators in the tuned model.
(TIF)

S2 Fig Model AUC score as a function of the maximum decision tree depth. This step was used during model hyperparameter tuning to select the maximum decision tree depth in the tuned model.
(TIF)

S3 Fig Percent correct classification as a function of the number of features included in the model. This step was used during model hyperparameter tuning to determine how many features to include in the tuned model.
(TIF)

S4 Fig Feature importance. This step was used during model hyperparameter tuning to select which features to include in the tuned model.
(TIF)

S5 Fig Tuned model accuracy. Confusion matrix of predictive accuracy of the final tuned model. Out-of-bag error was 0.021, while accuracy (% of predictions correct) was 97.9% and balanced accuracy (average accuracy per class) was 97.0%.
(TIF)

S1 File Code descriptions. Each script title is provided with a description of what it does and what the inputs and outputs are.
(PDF)
