# Supplementary material for: Scallop fishing activity characterization in Southern New England: Offshore wind demands and fisheries-dependent methods
Source: PLoS One. 2024 Nov 11;19(11):e0313197. doi: 10.1371/journal.pone.0313197 (PMC11554225; doi:10.1371/journal.pone.0313197)
Supplement: S1 Table — (PDF) [file pone.0313197.s001.pdf]

S1 Table. Data acquisition

Data	Source	Confidentiality	Description	Weaknesses for Characterizing Fishing Location
Automatic Information System (AIS)	https://marinecadastre.gov/ais/	Public	High resolution (~ 1 minute pings) location information for any vessel	Only required on vessels >65 ft.; fishing status unknown
Vessel Monitoring System (VMS)	Requested through NOAA's Office of Law Enforcement	Required a non-disclosure agreement. All published data products must meet the Rule of Three.	Location information for fishing vessels	Not all fisheries required to have; different reporting frequencies (generally 30 minutes or 60 minutes)
Vessel Trip Reports (VTR)	Obtained through the ACCSP Data Warehouse	Required confidential access and state and federal approvals. All published data products must meet the Rule of Three.	Self-reported effort info (location, gear used, catch, etc.)	Limited reporting based on statistical areas; poor spatiotemporal resolution due to low reporting frequency
Dealer Reports (landings)	Obtained through the ACCSP Data Warehouse	Required confidential access and state and federal approvals. All published data products must meet the Rule of Three.	Value, amount, and grade of seafood landed	No information about where seafood came from
Northeast Fisheries Observer Program (NEFOP)	Requested through the Northeast Fisheries Observer Program	Required confidential access and state and federal approvals. All published data products must meet the Rule of Three.	Highly reliable fisheries observer data on catch, bycatch, gear, location, etc.	Only certain fisheries have observer requirements and limited coverage by vessels
NOAA Greater Atlantic Regional Fisheries Office (GARFO) Permit Files	https://www.greateratlantic.fisheries.noaa.gov/public/public/web/NEROINET/aps/permits/data/index.html	Public	Permit information for all federally-permitted fishing vessels	N/A
Bathymetry	https://www.northeastoceandata.org/files/metadata/Themes/Bathymetry/Bathymetry.htm	Public	Raster of bathymetry at a resolution of 90 meters for New England from coastal regions to the edge of the continental shelf	N/A

Moon Phase	https://www.almanac.com/astronomy/moon/calendar	Public - data were compiled from monthly calendars	Daily moon phase as a percentage (100% = full moon, 0% = new moon)	N/A
US Federal Waters (excluding state)	https://marinecadastre.gov/data/	Public – data file was created using publicly available data	Created a shapefile by clipping State Waters from Federal Exclusive Economic Zone	N/A
