# Supplementary figures and images for: Scallop fishing activity characterization in Southern New England: Offshore wind demands and fisheries-dependent methods
Source: PLoS One. 2024 Nov 11;19(11):e0313197. doi: 10.1371/journal.pone.0313197 (PMC11554225; doi:10.1371/journal.pone.0313197)

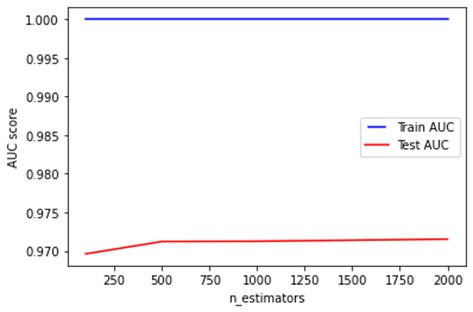

Supplement: S1 Fig — This step was used during model hyperparameter tuning to select the number of estimators in the tuned model. (TIF) [file pone.0313197.s002.tif]

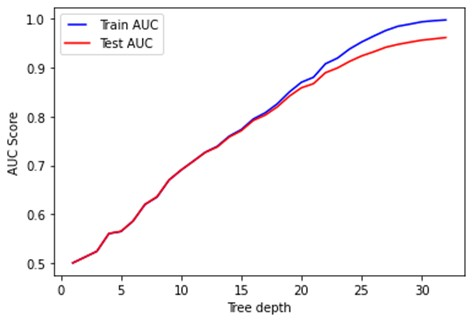

Supplement: S2 Fig — This step was used during model hyperparameter tuning to select the maximum decision tree depth in the tuned model. (TIF) [file pone.0313197.s003.tif]

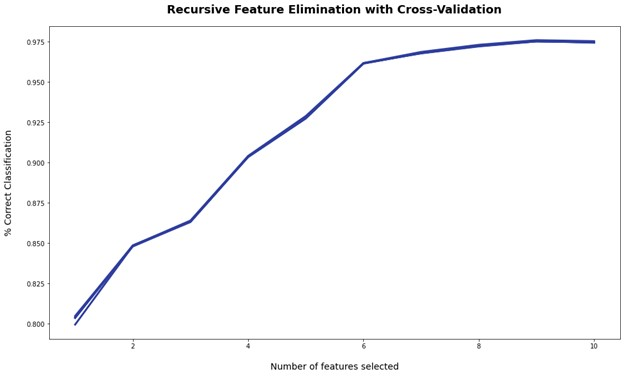

Supplement: S3 Fig — This step was used during model hyperparameter tuning to determine how many features to include in the tuned model. (TIF) [file pone.0313197.s004.tif]

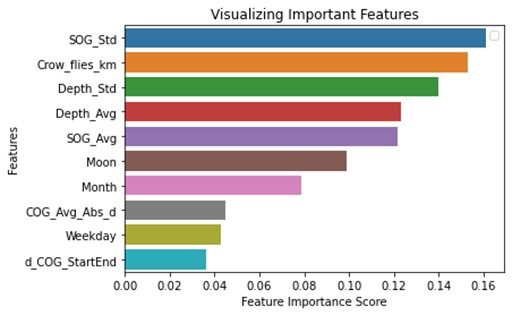

Supplement: S4 Fig — This step was used during model hyperparameter tuning to select which features to include in the tuned model. (TIF) [file pone.0313197.s005.tif]

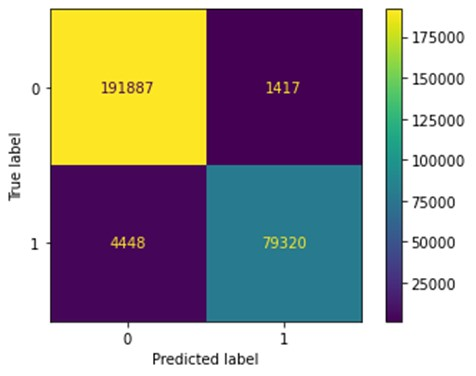

Supplement: S5 Fig — Confusion matrix of predictive accuracy of the final tuned model. Out-of-bag error was 0.021, while accuracy (% of predictions correct) was 97.9% and balanced accuracy (average accuracy per class) was 97.0%. (TIF) [file pone.0313197.s006.tif]
