# Supplementary material for: Scallop fishing activity characterization in Southern New England: Offshore wind demands and fisheries-dependent methods
Source: PLoS One. 2024 Nov 11;19(11):e0313197. doi: 10.1371/journal.pone.0313197 (PMC11554225; doi:10.1371/journal.pone.0313197)
Supplement: S1 File — Each script title is provided with a description of what it does and what the inputs and outputs are. (PDF) [file pone.0313197.s007.pdf]

Code (script) descriptions

These scripts are in the order that they should be run. Some scripts were also written to work on multiple FMPs/gear groupings.

All code is available in a GitHub repository: jlivermore-uri/Scallop_AIS

- Exploring_NEFOP_No_Loran_Bounds.ipynb
  - Initial script exploring NEFOP data and calculating tow lengths by gear
  - Data
    - Inputs:
      - NEFOP files (catch and trip tables)
    - Outputs:
      - Tow lengths table (gear_NEFOP_tow_lengths.csv)
- Merging_VMS_to_NEFOP.ipynb
  - Merges VMS files to NEFOP trips - key here is pulling in declaration code
  - Data
    - Inputs:
      - VMS (entire folder of csvs in ST19-001)
      - NEFOP trip files (riobtrip_time_formatted.csv)
    - Outputs: Merged NEFOP trip information with VMS declaration code (in VMS folder, VMS_NEFOP_alignment.csv and VMS_NEFOP_alignment_merged.csv)
- Training_Dataset_Generation.ipynb
  - Where the mega loop NEFOP merge with AIS happens
  - Should also drop all bad trips (where NEFOP fishing is not 0 or 1)
  - Data
    - Inputs:
      - AIS_GARFO_MATCHED_2015-2018.csv (just vessel and permit info)
      - NEFOP_CATCH_VES_SP_GEAR_noLoranBound.csv
      - riobhau03222021.csv
      - VMS_NEFOP_alignment.csv
      - All AIS monthly files between 2015 and 2018 (in AIS folder)
    - Outputs:
      - For each trip a csv will be saved in the Training Data folder titled either tripID_year_good.csv or tripID_year_bad.csv where good means all NEFOP fishing scores are 0s or 1s and bad means there are other numbers so the whole trip must be scrapped)
      - All files ending in *good.csv appended into all_training_data.csv

            - All good training data is also summarized by declaration code in training_data_Dec_Code_summary.csv
            - Also saved a NEFOP table: NEFOP_for_comparison.csv
- Remove_st_waters_add_bathy.R
    - Done in R for simplicity
    - Dropping state waters and pulling in bathymetry data for all NEFOP and AIS merged data
    - Data
        - Inputs:
            - Bathymetry.tif
            - Fed_Non_St_Waters.shp
            - Individual files for all good trips titled tripID_year_good.csv
        - Outputs:
            - all_training_data_training_datwBathy_noSt.csv
            - Individual files for all good trips titled tripID_year_bathy_no_state.csv
- Time_Adjustment_1min.ipynb
    - Converts AIS NEFOP merged data into 1 minute time increments using ffil and interpolation
    - Downsampled to 1 second intervals and then drops everything not on a 1 minute mark
    - Data
        - Inputs:
            - Individual files for all good trips titled tripID_year_bathy_no_state.csv
        - Outputs:
            - csv files for each trip titled tripID_year_1min_gear.csv
- Feature_Engineering_Scallop.ipynb
    - This is the script for scallop dredging gear - see the 3 subsequent scripts for the other gear types. Everything is the same within these except for the window lengths and gear files being used.
    - Actually creates the new features that we're interested in across 15-minute windows (window length can easily be adjusted)
        - SOG_Avg (average speed over ground)
        - SOG_Std (standard deviation of speed over ground)
        - Crow_flies_km (straight line distance from start to end locations)
        - Depth_Avg (average depth)
        - Depth_Std (standard deviation of depth)
        - Moon (moon light)
        - Month (month dummy)

        - COG_Avg_Abs_d (average of change in course over ground between consecutive points throughout the window)
        - Weekday (weekday dummy)
        - d_COG_StartEnd (change in course over ground start to end point)
    - Data
        - Inputs:
            - csv files for each trip titled tripID_year_1min_gear.csv
            - Moon_phase.csv
        - Outputs:
            - csv files for each trip titled tripID_year_prepped_1min_gear.csv
            - all_scallop_trained.csv
- Unseen_AIS_remove_st_waters_add_bathy.R
    - Done in R for simplicity
    - Dropping state waters and pulling in bathymetry data for all AIS data
    - Note: 2015-2017 required one loop and 2018 needed its own loop because formatting of the AIS files was different in 2018.
    - Data
        - Inputs:
            - Bathymetry.tif
            - Fed_Non_St_Waters.shp
            - Individual AIS date files titled AIS_YEAR_MO_DT.csv
        - Outputs:
            - Individual files for all AIS dates titled AIS_YEAR_MO_DT_bathy_no_state.csv
- Unseen_AIS_to_VMS.ipynb
    - Merges AIS to GARFO permit file by MMSI
    - Then merges AIS-GARFO to respective VMS data to pull in declaration code
        - Merges to VMS by creating a new AIS timestamp to the minute (drops seconds), then merging on permit number and timestamp to the VMS using an outer join (keeping all AIS). Then sorts by permit and time, uses forward fill to populate missing declaration codes and permit numbers for AIS points without a corresponding VMS point. Then drops all rows where permit number doesn't match (these would be created by the ffill where one permit switches to another - not due to improper earlier merge).
    - Inputs:
        - Individual files for all AIS dates titled AIS_YEAR_MO_DT_bathy_no_state.csv
        - AIS_GARFO_MATCHED_2015_2018.csv
        - Monthly VMS files titled YEAR_MO_wo_202126_file.csv
    - Outputs:

        - Individual day AIS files titled AIS_YEAR_MO_DT_w_dec.csv
- NEFOP_Check.ipynb
    - Building tables of seen vs unseen vessels and seen vessels with unseen trips
    - Inputs:
        - all_trawl_trained.csv
        - all_scallop_trained.csv
        - all_clam_trained.csv
        - all_gill_trained.csv
    - Outputs:
        - all_seen_trips.csv
        - All_seen_vessels.csv
- Unseen_AIS_1min_feat_eng.ipynb
    - Recalibrates AIS to standardized 1 min data and then engineers all features
    - Inputs: Individual day AIS files titled AIS_YEAR_MO_DT_w_dec.csv
    - Outputs:
        - Individual AIS day files titled AIS_YEAR_MO_DT_unseen_boats.csv
        - all_trawl_2015-2018.csv
        - all_gillnet_2015-2018.csv
        - all_scallop_2015-2018.csv
        - all_clam_2015-2018.csv
- RF_model_scallop.ipynb
    - Script to set up RF model for scallop data
    - Pulls in all scallop data and removes any rows with NA values in necessary columns (e.g., before a feature window has started) then runs RF model
    - Also tests OOS prediction accuracy on unseen vessels and on unseen trips for seen vessels
    - Inputs:
        - all_scallop_trained.csv
    - Outputs:
        - Confusion matrix plot
        - Accuracy (% correct predictions)
        - Balanced accuracy
        - Feature importance scores
        - Bar pot of feature importance
- RF_unseen_inference_scallop.ipynb - run on URI Cluster (Unity)
    - Script to conduct inference on unseen data based on tuned model from RF_model_scallop.ipynb
    - Inputs:
        - all_scallop_trained.csv
        - all_scallop_2015-2018.csv

    - Outputs:
        - scallop_unseen_inference.csv
- Data_cleanup_to_landings_cluster_scallop.ipynb
    - Combines seen and unseen data with either fishing predicted or known fishing from NEFOP.
    - Compares to VMS speed cutoff. Merges to landings data (evenly distributes dollars) after dropping state waters and non-fishing points.
    - Identifies trips to fall back to VMS or VTR for.
    - Inputs:
        - scallop_unseen_inference.csv
        - all_scallop_trained.csv
        - Monthly landings files
    - Outputs:
        - All_scallop_out.csv
            - This contains all AIS points (seen and unseen) after inference regarding fishing status.
        - scallop_pt_values_AIS.csv
            - This is just the fishing points from the AIS with the landings data evenly distributed across points within each trip.
        - Percentages of fishing points observed and all locations (fishing and non) observed
- Fallback_to_VMS-VTR_scallop.ipynb
    - Isolates scallop trips in VTR not covered by the AIS dataset already.
    - Then isolates all relevant VMS data for trips without AIS and distributes landings values evenly across VMS fishing points (based on speed filter).
    - Combines AIS and VMS into a single table of point locations with corresponding values.
    - Determines which trips are not addressed by AIS or VMS and saves a .csv containing that VTR data.
    - Calculates the number of trips covered by each of the 3 datasets by year.
    - Also generates table of VTRs by FMP (based on what they landed) that occurred within the study area. This can be used for the larger loop for all FMPs when we're there. This includes ALL trips in the VTR, not parsed out by VMS or AIS coverage yet.
    - Inputs:
        - VTR_2015.txt, VTR_2016.txt, VTR_2017.txt, VTR_2018.txt
        - all_land_2015-2018.csv
        - scallop_pt_values_AIS.csv (this is just AIS point values)
        - All_vms_2015-2018.csv (this was created inside this script but the creation code has been commented out so we can just read in this file now)

    - Outputs:
        - scallop_pt_values_AIS+VMS_datasets.csv
            - Contains all fishing points and corresponding values from landings for AIS and VMS only.
        - scallop_pts_uncovered_VTR.csv
            - Contains the remaining trip information that we need to address with VTR data.
        - ALL_FMP_vtr_trips.csv
            - Table of VTRs by FMP (based on what they landed) that occurred within the study area. This can be used for the larger loop for all FMPs when we're there. This includes ALL trips in the VTR, not parsed out by VMS or AIS coverage yet.
- VTR_compiling_scallop.r
    - Data were provided by NEFSC from their fishing footprints data product. We have a raster for each individual subtrip (IMGID) of a VTR for the Northeast (GARFO area). We now need to isolate only the data not already covered by AIS and VMS.
        - Data were provided as R .grd and .gri files so this step was conducted in R for simplicity.
    - Inputs:
        - scallop_pts_uncovered_VTR.csv
        - All 688,210 + rasters from NEFSC
        - scallop_ALL_vtr_trips.csv
    - Outputs:
        - Rasters for uncovered trips that we're using VTR to address
            - allYrsScallopVTR.tif
            - Individual rasters by year named: YEAR_ScallopVTR.tif
        - Rasters for all trips to use in final comparisons
            - allYrsALL_ScallopVTR.tif
            - Individual rasters by year named: YEAR_ALL_ScallopVTR.tif
- Mapping_AIS_VMS_scallop.r
    - Creates rasters of combined AIS and VMS in the same spatial reference as the VTR. Then combines all three to generate final outputs.
    - Also generates difference rasters between our product and the full VTR rasters, calculates the Moran's I of those difference rasters, and plots spatial autocorrelation.
    - Inputs:
        - scallop_pt_values_AIS+VMS_datasets.csv
        - Rasters for uncovered trips that we're using VTR to address
            - allYrsScallopVTR.tif

            - Individual rasters by year named: YEAR_ScallopVTR.tif
        - Rasters for all trips to use in final comparisons
            - allYrsALL_ScallopVTR.tif
            - Individual rasters by year named: YEAR_ALL_ScallopVTR.tif
    - Outputs:
        - scal_allYrsAisVmsRast.tif
        - Individual rasters by year named: YEAR_scal_AisVmsRast.tif
        - scal_allYrs_AISVMSVTR.tif
        - YEAR_AISVMSVTR.tif
- VMS_NEFOP_for_comparison.ipynb
    - Merges VMS to NEFOP trips for comparing accuracy with AIS models - this is for ALL FMPs
    - Inputs:
        - NEFOP tow data (NEFOP_for_comparison.csv)
        - All VMS data in annual folders
    - Outputs:
        - VMS_NEFOP_accuracy_check.csv
- Scallop_VTR_NEFOP_comparison.R
    - Compares accuracy of VTR rasters to NEFOP
        - Creates a raster of fishing vs. non-fishing based on NEFOP for each trip (does by making line between haul start and end locations and then rasterizes into a grid of 1s and 0s with size and resolution matching the corresponding VTR .grd file).
        - Then runs OLS regression for each trip and exports model results into a table.
    - Inputs:
        - NEFOP tow data (NEFOP_for_comparison.csv)
        - IMGID vs VTR Number lookup table
        - All raster VTR .grd files
    - Outputs:
        - Csv of OLS model outputs
